# Effects of Taurine-, Caffeine-, and Phosphatidylserine-Containing Supplementation Protocols on Physical and Cognitive Performance in Professional Male Football Players

**DOI:** 10.3390/nu18111684

**Published:** 2026-05-25

**Authors:** Krzysztof Mizera, Elżbieta Mizgała-Izworska, Justyna Mizera, Jan Mackiewicz

**Affiliations:** 1 Faculty of Medical Sciences and Health Sciences, Vizja University in Warsaw, 01-143 Warsaw, Poland; 2 Department of Family Medicine, Faculty of Medical Sciences, Silesian Medical University, 41-800 Zabrze, Poland; 3 Warsaw University of Medical and Technical Sciences, 02-366 Warsaw, Poland; 4 Doctoral School, Józef Piłsudski University of Physical Education in Warsaw, 00-968 Warsaw, Poland

**Keywords:** football, sports nutrition, dietary supplementation, caffeine, taurine, phosphatidylserine

## Abstract

**Background**: Nutritional supplementation is widely used to support physical and cognitive performance in football. However, evidence on multi-ingredient protocols combining taurine, caffeine, and phosphatidylserine (PS) remains limited in professional athletes. **Methods**: Eighty-one professional male football players (19–32 years) were randomly assigned to three groups (*n* = 27): placebo (P), taurine + caffeine (TC; 1500 mg taurine + 200 mg caffeine), and taurine + caffeine + PS (TCP; 1500 mg taurine + 150 mg caffeine + 300 mg PS) in a randomized, placebo-controlled, single-blind trial. Supplementation lasted 10 days, with a final dose administered 60 min before a standardized 105 min training session. Reaction time, sprint performance, GPS-derived variables, and technical/tactical indicators were assessed. Data were analyzed using ANOVA with post hoc tests, and pairwise comparisons were additionally adjusted using the Holm–Bonferroni correction due to the exploratory nature of the analysis. **Results**: Compared with placebo, the TCP group was associated with more favorable physical, cognitive, and selected game-related outcomes (*p* < 0.05). TCP was associated with higher locomotor performance (η^2^ = 0.13–0.20) and smaller fatigue-related declines in sprint performance (−18% vs. −34%) and speed (−10% vs. −19%) (η^2^ = 0.18–0.22). Reaction time and technical indicators, including passing accuracy (84% vs. 75%) and dribbling success (73% vs. 62%), were also improved. Higher coach-rated tactical performance scores were observed in TCP (η^2^ = 0.19–0.25). **Conclusions**: A short-term multi-ingredient protocol including taurine, caffeine, and PS may be associated with improved physical, cognitive, and selected game-related outcomes in professional football players. However, due to differences in caffeine dosage between groups, the independent effect of PS cannot be determined. Further double-blind studies are warranted. Given the exploratory nature of the analysis, the multiple assessed outcomes, and the partly subjective coach-rated tactical evaluations, the cognitive and tactical findings should be interpreted cautiously and regarded as preliminary rather than confirmatory evidence.

## 1. Introduction

Football is an intermittent high-intensity sport that requires the integration of physical, technical, and cognitive abilities. During a match, players typically cover distances of approximately 10–13 km, performing repeated sprints and technical actions while continuously processing information and responding to rapidly changing game situations. Therefore, cognitive functions such as attention, information processing speed, and reaction time play an important role in overall performance [1].

Cognitive performance may be impaired by physiological factors associated with prolonged exercise. Dehydration and exercise-induced stress can influence perceived fatigue as well as the ability to perform tasks requiring concentration and decision-making. In addition, fatigue has a central component (central fatigue), which is associated with the functioning of the central nervous system and the regulation of neurotransmission.

Caffeine is one of the most extensively studied ergogenic aids, with well-documented effects on both physical and cognitive performance. Systematic reviews indicate that caffeine ingestion may improve attention, alertness, and processing speed, particularly under conditions of fatigue [2]. Its primary mechanism of action involves antagonism of adenosine receptors and increased central nervous system stimulation. However, the effects of caffeine appear to depend on dose and individual sensitivity, and excessive stimulation may negatively affect cognitive control [3]. It has also been suggested that caffeine may partially counteract the negative effects of fatigue and sleep deprivation on reaction time and attention [4].

Taurine is a neuromodulatory compound that may influence neuronal excitability and central nervous system function. Available evidence suggests that its independent effects on cognitive performance are limited and somewhat inconsistent. However, when combined with caffeine, taurine may modify physiological responses, although the results reported in the literature remain inconclusive [5].

Phosphatidylserine (PS) is a phospholipid component of neuronal cell membranes and plays a role in maintaining their structural and functional integrity. Previous studies suggest that PS supplementation may reduce perceived fatigue and support exercise capacity, which could indicate its involvement in central mechanisms of fatigue [6]. Mechanistically, PS is involved in membrane fluidity and neurotransmission processes, which may be relevant for cognitive performance [7].

Furthermore, the physiological stress response, including activation of the hypothalamic–pituitary–adrenal (HPA) axis, has been shown to influence both cognitive function and fatigue perception [8]. Therefore, nutritional strategies that may modulate stress responses could potentially support cognitive performance during prolonged exercise.

The selection of the combined supplementation protocol was based on the complementary physiological roles of the included compounds. Caffeine is widely recognized for its ergogenic and cognitive effects, particularly in high-intensity and intermittent exercise. Taurine has been associated with neuromodulatory and fatigue-related processes, potentially supporting exercise performance and recovery. Phosphatidylserine has been investigated in relation to stress responses, cognitive function, and fatigue perception, although evidence in sport-specific contexts remains limited.

However, interpretation of multi-ingredient supplementation studies remains challenging, as interactions between compounds may result in additive or potentially synergistic effects that cannot be easily isolated within the present study design. In this context, it is not possible to distinguish whether the observed effects reflect additive or synergistic mechanisms between compounds.

Although phosphatidylserine has been linked to cognitive and stress-related outcomes, the available evidence in sport-specific settings remains limited, and its role in performance enhancement is not yet clearly established.

Despite growing interest in nutritional strategies supporting performance in football, the existing evidence remains limited and fragmented. Most previous studies have focused on single ergogenic compounds or simple combinations, with relatively little attention given to multi-ingredient supplementation protocols in intermittent high-intensity sports such as football.

Importantly, there is a lack of research examining the effects of combined taurine, caffeine, and phosphatidylserine supplementation in professional football players. Furthermore, previous studies have predominantly assessed isolated physical or cognitive outcomes, while evidence regarding technical-tactical performance and decision-making under fatigue conditions remains scarce.

Therefore, there is a clear need for studies that integrate physical, cognitive, and game-related performance measures in ecologically valid football-specific settings when evaluating multi-ingredient supplementation strategies. To our knowledge, no previous study has simultaneously examined these outcomes using this specific supplementation protocol in professional football players.

Furthermore, due to differences in caffeine dosage between the TC and TCP groups, the independent contribution of phosphatidylserine cannot be determined.

Therefore, the aim of the present study was to evaluate the effects of combined supplementation with caffeine, taurine, and phosphatidylserine on cognitive and physical performance, including reaction time, sprint ability, and fatigue-related parameters, in professional male football players.

We hypothesized that the TCP supplementation protocol would be associated with improvements in selected physical, cognitive, and game-related performance variables compared with TC and placebo conditions.

## 2. Materials and Methods

### 2.1. Study Design and Participants

The study was conducted in accordance with the Declaration of Helsinki. All participants provided written informed consent prior to participation. The study protocol was approved by the Ethics Committee of the University of Economics and Human Sciences in Warsaw (approval number: 01/11/2024; date of approval: 12 November 2024). The study was retrospectively registered at ClinicalTrials.gov (NCT07580248).

Randomization was performed using a computer-generated sequence, and the allocation list was prepared by an independent researcher not involved in data collection or analysis.

Eighty-one professional male football players from the 2nd Polish league were recruited. Although this sample size may be considered moderate, it represents a significant cohort of elite athletes. Access to professional league players is inherently limited due to strict club regulations and the high demands of professional training schedules, making this group a highly representative sample for elite-level sports science research. Training sessions were conducted on natural grass under similar environmental conditions and at comparable times of day.

Body composition was assessed using a bioelectrical impedance analyzer (InBody 180). Mean body mass was 77.0 ± 4.6 kg, and mean body height was 179 ± 4 cm.

Players trained approximately 1.5–2 h per day, typically 4–5 times per week, in addition to matches. All participants were healthy and reported no injuries or chronic conditions. None of the participants reported regular medication use, smoking, or alcohol consumption. Inclusion criteria included: professional male football players actively competing at the national level, regular participation in team training, and absence of injury at the time of the study.

Exclusion criteria included: current injury, chronic disease, medication use, smoking, and regular use of dietary supplements.

For 7 days prior to the study, participants refrained from caffeine and dietary supplements and followed a balanced diet (55–60% carbohydrates, 15–20% protein, 20–30% fat). However, dietary intake, sleep quality, and recovery status were not objectively monitored.

The study employed a randomized, placebo-controlled, single-blind design. Participants were blinded to group allocation.

No stratification by playing position was applied due to the team-based nature of the study.

### 2.2. Experimental Design and Supplementation

Participants were randomly assigned to three groups (*n* = 27 each) using a computer-generated randomization procedure. No stratification according to playing position or baseline characteristics was applied due to the relatively small sample size and team-based design. Allocation concealment was ensured by using coded group assignments that were not disclosed to the investigators responsible for participant allocation. Stratified randomization was not applied due to the relatively small sample size and team-based design.

Supplements were prepared and coded by an independent individual, and investigators conducting outcome assessments were not involved in group allocation.

The groups were as follows:

- Group P (placebo): lactose capsules;
- Group TC: taurine (1500 mg) + caffeine (200 mg);
- Group TCP: taurine (1500 mg) + caffeine (150 mg) + phosphatidylserine (300 mg/day for 10 days prior to testing).

All participants received supplementation for 10 days prior to testing. In Group P, participants ingested placebo tablets (lactose); in Group TC, taurine (1500 mg) and caffeine (200 mg) were administered; whereas in Group TCP, taurine (1500 mg), caffeine (150 mg), and phosphatidylserine (300 mg/day) were administered.

All capsules were identical in appearance, and participants were not informed about the type or dosage of supplements administered. Therefore, the study was conducted using a single-blind design, with elements of allocation concealment.

To minimize potential bias, a strict blinding protocol was implemented. While the study is primarily described as single-blind regarding the participants, it featured elements of double-blinding: the entire coaching staff responsible for tactical evaluations and one of the three primary investigators involved in data collection was blinded to the group assignments. Only two researchers, responsible for supplement preparation and distribution, had access to the allocation list.

Additionally, a final dose of the assigned supplementation (placebo, taurine + caffeine, or taurine + caffeine + PS) was administered 60 min before the training session.

It should be noted that the caffeine dose differed slightly between TC (200 mg) and TCP (150 mg), which may influence the interpretation of the specific effects of phosphatidylserine.

Supplement administration was supervised and recorded by the research staff to ensure compliance with the protocol.

### 2.3. Training Protocol

Participants completed a standardized 105 min football training session consisting of a 15 min warm-up followed by 90 min of football-specific training. GPS-derived performance analyses were performed exclusively during the 90 min football-specific training phase and did not include the warm-up period. The 90 min training phase was divided into two blocks to simulate match-related fatigue and repeated high-intensity efforts.

The session consisted of a 15 min standardized warm-up, followed by 15 min of technical drills, 30 min of match simulation, an additional 15 min of drills, and a final 30 min match simulation.

All sessions were performed at similar times of day under comparable environmental conditions.

Each player was equipped with a GPS device (Catapult Sports, Melbourne, Australia; 10 Hz GPS and 100 Hz accelerometer), positioned on the upper back.

Training load was standardized within the team training schedule; however, individual external and internal training loads prior to testing were not independently quantified.

### 2.4. Performance Monitoring

During training, external load and locomotor activity of the players were assessed. The following variables were recorded: total distance covered, running speed, high-speed running distance, number of sprints, accelerations, and decelerations.

High-speed running was defined as >19.8 km·h^−1^, and sprinting as >25.0 km·h^−1^. Accelerations and decelerations were defined using thresholds of ±2.5 m·s^−2^.

As the system incorporates a triaxial accelerometer, gyroscope, and magnetometer, a detailed characterization of movement patterns, including changes in direction and micro-movements, was possible. Total mechanical load was quantified using PlayerLoad, a composite variable derived from triaxial accelerometry.

The training session was additionally recorded using a drone, and the video footage was subsequently analyzed by the coaching staff.

Moreover, technical and tactical performance was assessed by the coaching staff based on standardized observational criteria derived from video recording. Scores were assigned by experienced coaches using predefined evaluation criteria. Evaluations were conducted independently, and final scores were determined by consensus.

The coaching staff consisted of a head coach and 3–4 assistant coaches. Evaluations were conducted independently, and final scores were determined by consensus.

Although this approach may still involve some degree of subjectivity, it reflects typical practice in football performance analysis. Inter-rater reliability was assessed using intraclass correlation coefficients (ICC), demonstrating high agreement across exploratory variables.

### 2.5. Psychomotor and Speed Testing

Reaction time was assessed using two independent methods. Visual reaction time was measured using a YB-1A reaction timer (Takei Scientific Instruments Co., Ltd., Niigata, Japan). Participants were instructed to respond as quickly as possible to a visual stimulus by pressing a response button. After a familiarization phase, a series of repeated trials was performed, and the final result was calculated as the mean of valid trials. The total testing time per participant was approximately 1–2 min.

Auditory reaction time was assessed using a computerized simple reaction time test implemented in PsychoPy software (version 2023.2, Open Science Tools Ltd., Nottingham, UK) on a standard laptop computer, with auditory stimuli delivered through over-ear headphones (e.g., Sony WH-1000XM4, Sony Corp., Tokyo, Japan). Participants responded by pressing a designated key on an external response device (Cedrus RB-740 response pad, Cedrus Corporation, San Pedro, CA, USA) following the presentation of a brief auditory stimulus delivered after a random foreperiod. After 3 familiarization trials, 10 valid trials were recorded, with interstimulus intervals randomized between 2 and 5 s. The final result was calculated as the mean of valid trials. The total testing time per participant was approximately 2 min.

All devices were calibrated prior to testing according to the manufacturers’ recommendations to ensure measurement accuracy and timing precision. The assessments were conducted in a dedicated, quiet testing room located at the stadium, under controlled environmental conditions. During testing, only one participant and two researchers were present in the room, while the remaining players waited outside (in the locker room) to minimize external distractions and ensure standardized testing conditions.

The applied protocol demonstrated acceptable reliability based on previous literature.

### 2.6. Statistical Analysis

The primary outcome was sprint performance (30 m time), while secondary outcomes included reaction time, GPS-derived variables, and technical performance measures. All reported values represent post-intervention outcomes obtained during the testing session following the supplementation period. Given the relatively large number of variables and pairwise comparisons, and consistent with the reviewer’s recommendation, the analysis is reported as exploratory in nature and all pairwise *p*-values were additionally adjusted for multiple comparisons. No a priori power analysis was performed; therefore, the study should be considered exploratory in nature. Data were analyzed using Statistica 13.0 (TIBCO Software Inc., Palo Alto, CA, USA), and the graphs were made using R software 4.3.2 (R Foundation for Statistical Computing, Vienna, Austria).

Normality was assessed using the Shapiro–Wilk test, and homogeneity of variances using Levene’s test.

Between-group differences were analyzed using one-way ANOVA, followed by Tukey’s post hoc test. In addition, all pairwise between-group comparisons were re-examined using independent-samples *t*-tests with the Holm–Bonferroni step-down correction applied within each variable family (three comparisons per variable).

Fatigue-related variables were analyzed using two-way repeated measures ANOVA (group × time). Percentage decline variables were calculated as: [(second half − first half)/first half × 100].

Effect sizes were calculated using partial eta squared (η^2^) for the omnibus ANOVA. For pairwise comparisons, between-group standardized mean differences were quantified using Cohen’s *d*, calculated as the difference in means divided by the pooled standard deviation, with the sign retained. The magnitude of d was interpreted using Cohen’s (1988) benchmarks: |d| < 0.20 negligible; 0.20–0.49 small; 0.50–0.79 medium; 0.80–1.19 large; 1.20–1.99 very large; ≥2.00 huge. Two-sided 95% confidence intervals (95% CI) are reported for all group means and for between-group mean differences. Statistical significance was set at *p* < 0.05.

Due to the relatively large number of assessed outcomes, multiple comparisons, and the exploratory nature of several variables, the possibility of inflated Type I error cannot be excluded; therefore, the findings should be interpreted with caution, and the interpretation of coach-rated tactical and selected technical outcomes should be tempered accordingly. The present analysis should therefore be considered exploratory and hypothesis-generating. Accordingly, differences in cognitive and coach-rated tactical outcomes should be interpreted cautiously and confirmed in adequately powered, double-blind studies with pre-registered hypotheses.

## 3. Results

All results are presented as post-intervention values measured on Day 10, unless otherwise stated.

### 3.1. Psychomotor and Sprint Performance

**Figure 1:**
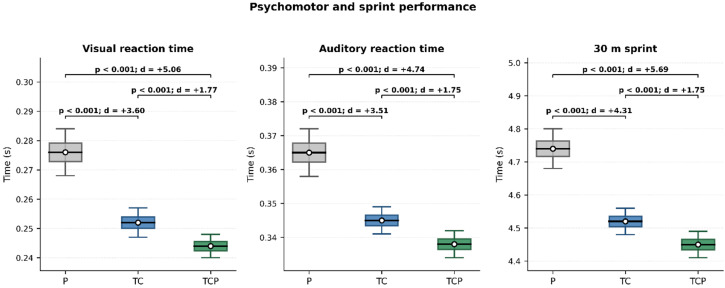
Visual reaction time, auditory reaction time and 30 m sprint performance in the placebo (P), taurine + caffeine (TC), and taurine + caffeine + phosphatidylserine (TCP) groups. Boxes show the 95% confidence interval of the group mean; whiskers indicate mean ± SD; the inner horizontal line and central marker denote the group mean. Pairwise between-group comparisons are annotated with Holm–Bonferroni-adjusted *p*-values and Cohen’s *d* (with sign retained) instead of asterisks; bold annotations indicate *p*_adj < 0.05. P, placebo; TC, taurine + caffeine; TCP, taurine + caffeine + phosphatidylserine; SD, standard deviation; 95% CI, 95% confidence interval.

Psychomotor and sprint performance variables are presented in Figure 1 and Table 1.

Values are presented as mean ± SD with 95% confidence intervals for group means provided in brackets. Statistically significant differences between groups were observed for all variables (*p* < 0.001) with very large to huge effect sizes for all pairwise comparisons (|d| = 1.75–5.69; all *p*_adj < 0.001 after Holm–Bonferroni correction; see pairwise table below). The TCP group consistently achieved the best performance, whereas the placebo group showed the lowest values. Different superscripts (a, b, c) within the same row indicate statistically significant differences between groups based on Tukey’s post hoc test (*p* < 0.05).

### 3.2. GPS-Derived Performance Variables

**Figure 2:**
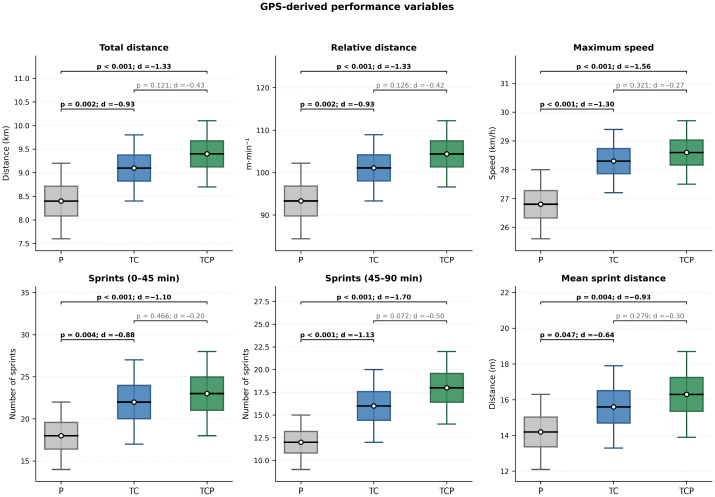
GPS-derived performance variables during the football-specific training session. Boxes represent 95% CI of the group mean, whiskers mean ± SD, and the inner horizontal line/central marker the group mean. Pairwise comparisons are annotated with Holm–Bonferroni-adjusted *p*-values and Cohen’s *d* (instead of asterisks); bold annotations indicate *p*_adj < 0.05. Performance analyses were performed during the 90 min football-specific training phase and did not include the warm-up period. P, placebo; TC, taurine + caffeine; TCP, taurine + caffeine + phosphatidylserine; GPS, global positioning system.

The total session lasted 105 min, including a standardized 15 min warm-up. GPS-derived performance variables were analyzed during the main 90 min football-specific training phase, divided into two periods: 0–45 min and 45–90 min. GPS-derived performance variables are presented in Figure 2 and Table 2.

Values are presented as mean ± SD with 95% CI of group means in brackets. Statistically significant differences between groups were observed for total distance covered, relative distance, maximal speed, and selected sprint-related variables (*p* < 0.05). Higher values were generally observed in the TCP group compared with the placebo. After Holm–Bonferroni correction, P vs. TCP differences remained significant for all GPS variables with large to very large effect sizes (|d| = 0.93–1.70), and P vs. TC differences remained significant with medium to very large effects (|d| = 0.64–1.30). TC vs. TCP differences did not survive correction for any GPS variable (all *p*_adj ≥ 0.072), with small effects (|d| = 0.20–0.50). This pattern indicates that the principal performance gain attributable to phosphatidylserine addition over taurine + caffeine alone, for these GPS-derived metrics, is small and statistically non-significant. * indicates adjusted *p* < 0.05.

### 3.3. Fatigue-Related Parameters

**Figure 3:**
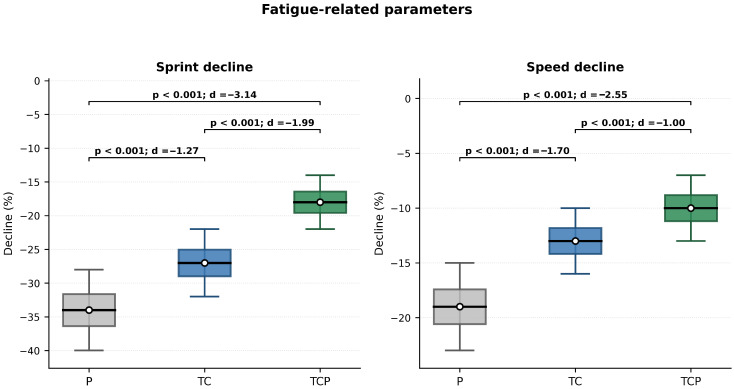
Fatigue-related parameters during the football-specific training session. Boxes represent 95% CI of the group mean; whiskers indicate mean ± SD; the inner horizontal line/central marker denotes the group mean. Pairwise comparisons are annotated with Holm–Bonferroni-adjusted *p*-values and Cohen’s *d* (instead of asterisks); bold annotations indicate *p*_adj < 0.05. Sprint decline and speed decline were calculated as the percentage change between the first and second halves of the training session: [(second half − first half)/first half × 100 P, placebo; TC, taurine + caffeine; TCP, taurine + caffeine + phosphatidylserine.

Fatigue-related decline was calculated as the percentage change between the first and second halves of the main 90 min football-specific training phase using the formula: [(second half − first half)/first half × 100]. Fatigue-related parameters are presented in Figure 3 and Table 3.

Values are presented as mean ± SD, with 95% CI of group means in brackets. A significant group × time interaction was observed for fatigue-related variables (*p* < 0.001). Lower percentage declines in sprint and speed performance were observed in the TCP group compared with the placebo. All three pairwise comparisons retained significance after Holm–Bonferroni correction for both sprint decline (all *p*_adj < 0.001; |d| = 1.27–3.14) and speed decline (all *p*_adj < 0.001; |d| = 1.00–2.55), with the largest effect for P vs. TCP. The pattern is consistent with a fatigue-attenuating effect of TCP supplementation that exceeds taurine + caffeine alone, particularly for the sprint decline outcome. Different superscripts (a, b, c) within the same row indicate statistically significant differences between groups based on Tukey’s post hoc test (*p* < 0.05). * indicates *p*_adj < 0.05.

### 3.4. Technical and Tactical Performance

**Figure 4:**
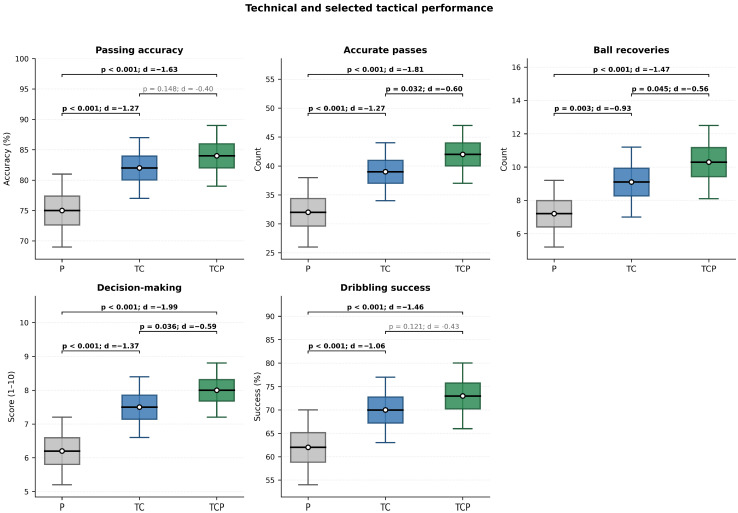
Technical and selected tactical performance variables during the football-specific training session. Boxes represent 95% CI of the group mean; whiskers indicate mean ± SD. Pairwise comparisons are annotated with Holm–Bonferroni-adjusted *p*-values and Cohen’s *d* (instead of asterisks); bold annotations indicate *p*_adj < 0.05. Variables were assessed using standardized video-based observational analysis. Passing accuracy and dribbling success are expressed as percentages, accurate passes and ball recoveries as counts, and decision-making as a coach-rated score on a 1–10 scale, where higher scores indicate better performance. P, placebo; TC, taurine + caffeine; TCP, taurine + caffeine + phosphatidylserine.

Technical and tactical performance variables assessed by the coaching staff are presented in Figure 4 and Table 4.

Values are presented as mean ± SD, with 95% CI of group means in brackets. Statistically significant differences between groups were observed for all variables (*p* < 0.01, η^2^ = 0.15–0.30). Technical variables were assessed using video-based observational analysis. Passing accuracy and dribbling success are expressed as percentages, accurate passes and ball recoveries as counts, and decision-making as a coach-rated score on a 1–10 scale. After Holm–Bonferroni correction, P vs. TCP differences remained significant for all five variables with very large effect sizes (|d| = 1.46–1.99); P vs. TC differences remained significant with large to very large effects (|d| = 1.06–1.37). TC vs. TCP differences survived correction for accurate passes, ball recoveries and decision-making with medium effects (|d| = 0.56–0.60; *p*_adj < 0.05), but not for passing accuracy or dribbling success (*p*_adj = 0.121–0.148). The cognitively loaded outcomes (decision-making, accurate passes) were interpreted with the caution recommended by the reviewer. Different superscripts (a, b, c) within the same row indicate statistically significant differences between groups based on Tukey’s post hoc test (*p* < 0.05). * indicates *p*_adj < 0.05.

### 3.5. Technical and Tactical Performance (Coaches’ Evaluation)

**Figure 5:**
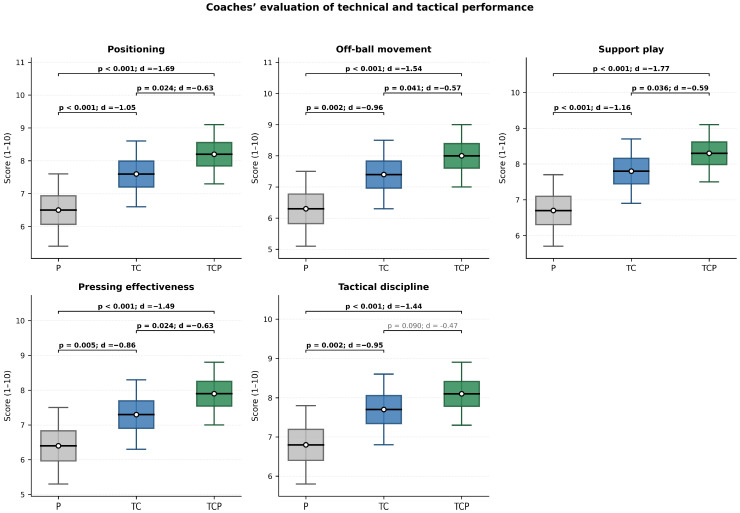
Coaches’ evaluation of technical and tactical performance variables during the football-specific training session. Boxes represent 95% CI of the group mean; whiskers indicate mean ± SD. Pairwise comparisons are annotated with Holm–Bonferroni-adjusted *p*-values and Cohen’s *d* (instead of asterisks); bold annotations indicate *p*_adj < 0.05. Variables were assessed using standardized observational criteria based on video recordings and expressed on a 1–10 scale, where higher scores indicated better performance. P, placebo; TC, taurine + caffeine; TCP, taurine + caffeine + phosphatidylserine.

Technical and tactical performance variables assessed by the coaching staff are presented in Figure 5 and Table 5.

Values are presented as mean ± SD, with 95% CI of group means in brackets. Statistically significant differences between groups were observed for all coach-rated variables (*p* < 0.01, η^2^ = 0.19–0.25). Variables were assessed using standardized observational criteria based on video recordings and expressed on a 1–10 scale, where higher scores indicate better performance. After Holm–Bonferroni correction, P vs. TCP comparisons remained significant for every coach-rated variable with very large effects (|d| = 1.44–1.77). P vs. TC differences also retained significance with large effects (|d| = 0.86–1.16). TC vs. TCP differences were significant for positioning, off-ball movement, support play and pressing effectiveness with medium effects (|d| = 0.57–0.63; *p*_adj = 0.024–0.041), but did not survive correction for tactical discipline (*p*_adj = 0.090; |d| = 0.47). Because the coach-rated tactical scores are inherently subjective, the interpretation of differences—particularly the TC vs. TCP comparisons—is reported with the caution emphasized by the reviewer. Higher mean scores for positioning, off-ball movement, and support play were observed in the TCP group. * indicates *p*_adj < 0.05.

### 3.6. Summary of Results

The TCP group showed more favorable post-intervention values across selected physical, cognitive, and game-related performance variables compared with placebo and, for several outcomes, compared with TC. The pattern of effect sizes and Holm–Bonferroni-adjusted *p*-values supports a graded interpretation: P vs. TCP differences were consistently the largest (mostly very large to huge Cohen’s *d*) and survived multiple-comparison correction for every assessed variable, whereas TC vs. TCP differences were generally smaller and survived correction primarily for fatigue-related outcomes and most coach-rated tactical scores.

For GPS-derived metrics, group differences were observed for total distance, relative distance, sprint activity, maximal speed, and mean sprint distance (η^2^ = 0.13–0.20). The TCP group showed higher values than placebo, with the largest differences observed during the second half of the main training phase (P vs. TCP: |d| = 0.93–1.70, all *p*_adj < 0.05). The added benefit of phosphatidylserine over taurine + caffeine for GPS metrics was small and statistically non-significant after correction (TC vs. TCP: |d| = 0.20–0.50, *p*_adj = 0.072–0.466).

Fatigue-related decline was smaller in the TCP group than in the placebo for sprint decline (−18% vs. −34%) and speed decline (−10% vs. −19%) (η^2^ = 0.18–0.22). All pairwise contrasts for fatigue variables remained significant after Holm–Bonferroni correction (|d| = 1.00–3.14, all *p*_adj < 0.001), supporting a robust effect on within-session fatigue attenuation.

Reaction time values were lower in the supplemented groups, with the lowest values observed in TCP (P vs. TCP: |d| = 4.74–5.06, *p*_adj < 0.001; TC vs. TCP: |d| = 1.75–1.77, *p*_adj < 0.001). Technical performance variables also showed higher values in TCP, including passing accuracy (84% vs. 75%), number of accurate passes (42 vs. 32), and dribbling success (73% vs. 62%) (all P vs. TCP *p*_adj < 0.001; |d| = 1.46–1.99).

Coach-rated tactical variables showed higher scores in TCP for positioning, off-ball movement, and support play (η^2^ = 0.19–0.25) with most TC vs. TCP contrasts also retaining significance after correction (|d| ≈ 0.57–0.63, *p*_adj = 0.024–0.041), except for tactical discipline (*p*_adj = 0.090). Given the subjective nature of the coach-rated scale, these results are interpreted with appropriate caution.

Overall, the findings indicate that the TCP supplementation protocol was associated with more favorable performance-related outcomes under football-specific training conditions, with the effect being most consistent and largest for fatigue-related, reaction-time and core technical/tactical outcomes (large-to-huge Cohen’s *d* that survive Holm–Bonferroni correction), and smaller and less robust for the incremental benefit of phosphatidylserine over taurine + caffeine on GPS-derived load metrics. These findings should be interpreted with caution due to the exploratory nature of the analysis, the number of assessed outcomes, and the subjective component of the cognitive (reaction time) and tactical (coach-rated) evaluations.

## 4. Discussion

This study examined the effects of a 10-day supplementation protocol including caffeine, taurine, and phosphatidylserine (TCP) on physical and cognitive performance in professional soccer players. The results indicate that the TCP protocol was associated with more favorable physical and cognitive performance outcomes compared with both taurine + caffeine (TC) and placebo. To the best of our knowledge, this is one of the first studies to investigate a multi-ingredient supplementation protocol including phosphatidylserine in the context of intermittent high-intensity sport.

### 4.1. Potential Combined Effects of the Supplement Components

The more favorable performance and cognitive outcomes observed in the TCP group compared to the TC group may reflect the combined effects of the supplemented compounds; however, specific interactions between compounds cannot be determined based on the present study design.

Caffeine is a well-established ergogenic aid that may delay fatigue and enhance central nervous system activation through adenosine receptor antagonism [9,10]. Taurine has been associated with calcium handling and membrane stabilization, which may support muscle function during repeated high-intensity efforts [11,12]. Previous studies suggest that taurine may support muscle function and exercise performance, potentially through mechanisms related to calcium handling and muscle contractility [13]. High doses of stimulants may also influence activation of the hypothalamic–pituitary–adrenal (HPA) axis, potentially affecting stress responses [14].

Phosphatidylserine is a key phospholipid component of neuronal membranes and is associated with processes related to membrane stability and neurotransmission. It has also been reported to play a role in maintaining membrane fluidity and supporting neuronal membrane function; however, these mechanisms were not directly assessed in the present study [15].

Some studies suggest that phosphatidylserine supplementation may influence stress-related responses, including modulation of post-exercise adrenocorticotropic hormone (ACTH) and cortisol levels [16]. However, in the context of the present study, its specific contribution cannot be directly determined due to the study design.

Previous studies investigating combinations of cognitive-support supplements with caffeine have reported mixed effects on attention and cognitive performance in sport-related settings [17]. Overall, while the inclusion of phosphatidylserine may have contributed to the observed outcomes, its specific role and underlying mechanisms remain uncertain and warrant further investigation in controlled studies [18]. Recent systematic reviews suggest that phosphatidylserine supplementation may have potential benefits for cognitive function, exercise capacity, and selected aspects of athletic performance; however, the available evidence remains limited and inconsistent across studies [19].

The proposed physiological mechanisms discussed above remain hypothetical and were not directly evaluated in the present study.

### 4.2. Cognitive and Technical Performance

Football requires cognitive functions in concert with motor skills to monitor the environment and make choices during play [20]. In this study, the TCP group’s visual and auditory reaction times were not only shorter, but also increased passing accuracy and dribbling efficiency (84% vs. 75% and 73% vs. 62%), respectively. These findings may reflect differences in cognitive processing during football-specific tasks [21]; however, these pathways were not directly assessed in the present study. The present findings are partially consistent with previous studies examining combined caffeine and taurine supplementation in elite athletes. Ozan et al. reported that co-ingestion of caffeine and taurine was associated with improvements in reaction time, agility, balance, and anaerobic performance following fatigue-inducing exercise protocols in elite boxers. In particular, combined supplementation improved neutral reaction time compared with isolated caffeine, taurine, and placebo conditions. However, differences in sport characteristics, exercise protocols, and supplementation strategies should be considered when interpreting these findings [12].

Research on caffeine usage in soccer is somewhat limited. Some studies report improvements in accuracy [22], whereas others suggest increased impulsivity and impaired decision-making [23]. Recent literature indicates that caffeine supplementation may improve cognitive and physical performance in high-intensity sports, particularly in domains related to attention, reaction time, executive function, and fatigue resistance. Such effects may be especially relevant in intermittent sports requiring rapid perception–decision–action processes [5]. These findings may indicate a potential association between the TCP protocol and faster information processing during football-specific tasks [24]. It should be emphasized that the cognitive and technical interpretations presented above are exploratory. Reaction-time and decision-making outcomes were obtained in a sport-specific setting in which the combined effects of caffeine, taurine, and phosphatidylserine could not be separated within the present single-blind, unequal-caffeine design. The magnitude of the observed reaction-time differences exceeded that typically reported for isolated caffeine or taurine supplementation, suggesting the possibility of expectancy-related or order-related bias. Moreover, the technical indicators and coach-rated tactical scores included a partly subjective component and were assessed during a single training session, limiting generalizability. Therefore, these findings should be regarded as preliminary and confirmed in adequately powered, double-blind trials with matched caffeine doses.

### 4.3. Central Fatigue and Maintaining Intensity in the Second Half

One of the key performance challenges in soccer is the decline in repeated-sprint performance in the second half of a match [25]. During the second half of the training session, the TCP group presented with only an 18% decline in sprint performance as compared to 34% in the placebo group. This advantage is much larger than caffeine-induced performance reduction, which is often attenuated during the last minutes of a match [26,27]. These findings may reflect differences in fatigue-related responses and neuromuscular performance during repeated high-intensity efforts. Previous literature has suggested that phosphatidylserine may support central nervous system function under stress conditions; however, this was not directly assessed in the present study [19]. Taurine has been suggested to support muscle function by stabilizing sarcoplasmic membranes and modulating calcium handling [28]. Previous studies suggest that such mechanisms may support the maintenance of motor output during fatigue; however, in the present study, the combined supplementation may have contributed to sustaining neuromuscular performance despite increasing fatigue [29]. Recent studies investigating taurine and caffeine supplementation in football players reported that caffeine, as well as combined taurine and caffeine supplementation, prolonged time to exhaustion and improved selected cognitive outcomes during exercise; however, no significant improvements were observed in subsequent repeated sprint performance [30]. Similar findings have also been reported in elite athletes, where acute caffeine ingestion was associated with improvements in sport-specific cognitive and technical performance during high-intensity exercise conditions, including faster reaction time and improved task execution [31]. Mor et al. reported that acute caffeine supplementation enhanced anaerobic capacity and selected lower-limb performance measures in female soccer players, although no significant improvements were observed in agility, jump performance, or ball speed [32]. Previous literature suggests that mental fatigue may impair cognitive and technical performance in soccer players, particularly during prolonged intermittent exercise. Reported effects include reductions in decision-making performance, technical skill execution, and psychophysiological responses during soccer-specific tasks [3]. Recent sport-specific evidence further indicates that repeated multidirectional sprint protocols may simultaneously induce neuromuscular and cognitive fatigue responses in elite soccer players, supporting the concept that cognitive and physical fatigue may interact during football-specific exercise conditions [1].

### 4.4. Validity of Coaching Assessments

The validity of coaching assessments is increasingly recognized in sports science literature, particularly for enhancing ecological validity and better reflecting real training and match conditions [33]. Coach-rated outcomes were generally consistent with objective performance measures; however, due to their subjective nature and the lack of formal inter-rater reliability assessment, they should be interpreted with caution.

Higher coach-rated positioning and decision-making scores were observed in the TCP group under fatigue conditions. These factors are considered critical determinants of performance in professional football [34].

### 4.5. Limitations and Future Research Directions

The present study has several limitations that should be acknowledged. First, a single-blind design was used, and investigators were not blinded to group allocation. Although several outcomes were objectively measured, a double-blind design would further reduce the risk of expectancy-related bias [35].

Second, some outcomes—particularly technical and tactical performance—were based on coaching assessments and therefore remain partly subjective. However, these evaluations were supported by systematic analysis of video recordings and GPS-derived data, allowing coaches to assess technical and tactical aspects in a structured and context-specific manner. Although consensus scoring was applied, inter-rater reliability was not formally assessed, and future studies should incorporate fully objective performance analysis methods, such as advanced GPS and automated video tracking systems [36].

Third, no biochemical markers (e.g., cortisol, lactate) were measured, which limits the ability to directly verify the underlying physiological mechanisms.

Fourth, no a priori sample size calculation was performed. However, it should be noted that the recruitment of 81 professional league players is a considerable strength, as access to high-level athletes presenting a professional standard is often restricted, which typically results in much smaller sample sizes in sports nutrition literature. Finally, the unequal caffeine dosage between TC and TCP limits the ability to isolate the independent effects of phosphatidylserine. In addition, the formal inter-rater reliability of the coach-based technical and tactical evaluations was not assessed. Therefore, the findings should be interpreted as preliminary and hypothesis-generating.

## 5. Conclusions

The present findings suggest that a multi-ingredient supplementation protocol containing caffeine, taurine, and phosphatidylserine was associated with more favorable physical, cognitive, and selected game-related performance measures in professional football players. These findings support further investigation of combined nutritional strategies in intermittent high-intensity sports. However, because caffeine dosage differed between the active treatment groups, the independent contribution of phosphatidylserine cannot be determined.

Therefore, the practical application of this supplementation strategy should be considered preliminary. Further double-blind, pre-registered studies using matched supplementation protocols and objective performance measures are needed to confirm these findings and to better understand the observed performance-related effects.

Because the present analysis was exploratory and included partly subjective and multiple-comparison outcomes, the cognitive and tactical findings should be interpreted cautiously. These results should be considered preliminary and confirmed in adequately powered, fully double-blind trials with matched caffeine doses.

## Figures and Tables

**Table 1:** (**a**) Psychomotor and sprint performance (mean ± SD; 95% CI; ANOVA *p*-value; partial η^2^). (**b**) Pairwise between-group comparisons (Holm–Bonferroni-adjusted) for psychomotor and sprint variables.

(**a**)					
**Parameter**	**P (*n* = 27) M ± SD [95% CI]**	**TC (*n* = 27) M ± SD [95% CI]**	**TCP (*n* = 27) M ± SD [95% CI]**	***p* (ANOVA)**	**η^2^**
Visual reaction time (s)	0.276 ± 0.008 * [0.273, 0.279]*	0.252 ± 0.005 * [0.250, 0.254]*	0.244 ± 0.004 * [0.242, 0.246]*	<0.001	0.34
Auditory reaction time (s)	0.365 ± 0.007 * [0.362, 0.368]*	0.345 ± 0.004 * [0.343, 0.347]*	0.338 ± 0.004 * [0.336, 0.340]*	<0.001	0.29
30 m sprint (s)	4.74 ± 0.06 * [4.72, 4.76]*	4.52 ± 0.04 * [4.50, 4.54]*	4.45 ± 0.04 * [4.43, 4.47]*	<0.001	0.36
(**b**)					
**Parameter**	**Comparison**	**Mean Diff. [95% CI]**	**Cohen’s *d* (Mag.)**	***p* (Holm–Bonferroni)**	
Visual reaction time (s)	P vs. TC	+0.024 [0.020, 0.028]	+3.60 (huge)	<0.001 *	
	P vs. TCP	+0.032 [0.029, 0.035]	+5.06 (huge)	<0.001 *	
	TC vs. TCP	+0.008 [0.006, 0.010]	+1.77 (v.large)	<0.001 *	
Auditory reaction time (s)	P vs. TC	+0.020 [0.017, 0.023]	+3.51 (huge)	<0.001 *	
	P vs. TCP	+0.027 [0.024, 0.030]	+4.74 (huge)	<0.001 *	
	TC vs. TCP	+0.007 [0.005, 0.009]	+1.75 (v.large)	<0.001 *	
30 m sprint (s)	P vs. TC	+0.22 [0.19, 0.25]	+4.31 (huge)	<0.001 *	
	P vs. TCP	+0.29 [0.26, 0.32]	+5.69 (huge)	<0.001 *	
	TC vs. TCP	+0.07 [0.05, 0.09]	+1.75 (v.large)	<0.001 *	

Abbreviations: TC, taurine + caffeine; TCP, taurine + caffeine + phosphatidylserine; SD, standard deviation; η^2^, partial eta squared; M, mean; 95% CI, 95% confidence interval. Asterisk (*) indicates *p*_adj < 0.05. Magnitude labels: negl. = negligible (|d| < 0.20); small (0.20–0.49); med. = medium (0.50–0.79); large (0.80–1.19); v.large = very large (1.20–1.99); huge (≥2.00).

**Table 2:** (**a**) GPS-derived performance variables (mean ± SD; 95% CI; ANOVA *p*-value; partial η^2^). (**b**) Pairwise between-group comparisons (Holm–Bonferroni-adjusted) for GPS-derived performance variables. The Abbreviations, Magnitude labels and Asterisk (*) explanations prefer to Table 1 footer.

(**a**)					
**Parameter**	**P (*n* = 27) M ± SD [95% CI]**	**TC (*n* = 27) M ± SD [95% CI]**	**TCP (*n* = 27) M ± SD [95% CI]**	***p* (ANOVA)**	**η^2^**
Total distance (km)	8.40 ± 0.80 * [8.08, 8.72]*	9.10 ± 0.70 * [8.82, 9.38]*	9.40 ± 0.70 * [9.12, 9.68]*	<0.01	0.18
Distance (m·min^−1^)	93.3 ± 8.9 * [89.8, 96.8]*	101.1 ± 7.8 * [98.0, 104.2]*	104.4 ± 7.8 * [101.3, 107.5]*	<0.01	0.20
Sprints (0–45 min)	18.0 ± 4.0 * [16.4, 19.6]*	22.0 ± 5.0 * [20.0, 24.0]*	23.0 ± 5.0 * [21.0, 25.0]*	<0.05	0.14
Sprints (45–90 min)	12.0 ± 3.0 * [10.8, 13.2]*	16.0 ± 4.0 * [14.4, 17.6]*	18.0 ± 4.0 * [16.4, 19.6]*	<0.01	0.19
Max speed (km/h)	26.8 ± 1.2 * [26.3, 27.3]*	28.3 ± 1.1 * [27.9, 28.7]*	28.6 ± 1.1 * [28.2, 29.0]*	<0.01	0.17
Mean sprint distance (m)	14.2 ± 2.1 * [13.4, 15.0]*	15.6 ± 2.3 * [14.7, 16.5]*	16.3 ± 2.4 * [15.4, 17.2]*	<0.05	0.13
(**b**)					
**Parameter**	**Comparison**	**Mean Diff. [95% CI]**	**Cohen’s *d* (Mag.)**	***p* (Holm–Bonferroni)**	
Total distance (km)	P vs. TC	−0.70 [−1.11, −0.29]	−0.93 (large)	0.002 *	
	P vs. TCP	−1.00 [−1.41, −0.59]	−1.33 (v.large)	<0.001 *	
	TC vs. TCP	−0.30 [−0.68, 0.08]	−0.43 (small)	0.121	
Distance (m·min^−1^)	P vs. TC	−7.8 [−12.4, −3.2]	−0.93 (large)	0.002 *	
	P vs. TCP	−11.1 [−15.7, −6.5]	−1.33 (v.large)	<0.001 *	
	TC vs. TCP	−3.3 [−7.6, 1.0]	−0.42 (small)	0.126	
Sprints (0–45 min)	P vs. TC	−4.0 [−6.5, −1.5]	−0.88 (large)	0.004 *	
	P vs. TCP	−5.0 [−7.5, −2.5]	−1.10 (large)	<0.001 *	
	TC vs. TCP	−1.0 [−3.7, 1.7]	−0.20 (small)	0.466	
Sprints (45–90 min)	P vs. TC	−4.0 [−5.9, −2.1]	−1.13 (large)	<0.001 *	
	P vs. TCP	−6.0 [−7.9, −4.1]	−1.70 (v.large)	<0.001 *	
	TC vs. TCP	−2.0 [−4.2, 0.2]	−0.50 (med.)	0.072	
Max speed (km/h)	P vs. TC	−1.5 [−2.1, −0.9]	−1.30 (v.large)	<0.001 *	
	P vs. TCP	−1.8 [−2.4, −1.2]	−1.56 (v.large)	<0.001 *	
	TC vs. TCP	−0.3 [−0.9, 0.3]	−0.27 (small)	0.321	
Mean sprint distance (m)	P vs. TC	−1.4 [−2.6, −0.2]	−0.64 (med.)	0.047 *	
	P vs. TCP	−2.1 [−3.3, −0.9]	−0.93 (large)	0.004 *	
	TC vs. TCP	−0.7 [−2.0, 0.6]	−0.30 (small)	0.279	

**Table 3:** (**a**) Fatigue-related parameters (mean ± SD; 95% CI; ANOVA *p*-value; partial η^2^). (**b**) Pairwise between-group comparisons (Holm–Bonferroni-adjusted) for fatigue-related parameters. The Abbreviations, Magnitude labels and Asterisk (*) explanations prefer to Table 1 footer.

(**a**)					
**Parameter**	**P (*n* = 27) M ± SD [95% CI]**	**TC (*n* = 27) M ± SD [95% CI]**	**TCP (*n* = 27) M ± SD [95% CI]**	***p* (ANOVA)**	**η^2^**
Sprint decline (%)	−34.0 ± 6.0 * [−36.4, −31.6]*	−27.0 ± 5.0 * [−29.0, −25.0]*	−18.0 ± 4.0 * [−19.6, −16.4]*	<0.001	0.22
Speed decline (%)	−19.0 ± 4.0 * [−20.6, −17.4]*	−13.0 ± 3.0 * [−14.2, −11.8]*	−10.0 ± 3.0 * [−11.2, −8.8]*	<0.001	0.18
(**b**)					
**Parameter**	**Comparison**	**Mean Diff. [95% CI]**	**Cohen’s *d* (Mag.)**	***p* (Holm–Bonferroni)**	
Sprint decline (%)	P vs. TC	−7.0 [−10.0, −4.0]	−1.27 (v.large)	<0.001 *	
	P vs. TCP	−16.0 [−18.8, −13.2]	−3.14 (huge)	<0.001 *	
	TC vs. TCP	−9.0 [−11.5, −6.5]	−1.99 (v.large)	<0.001 *	
Speed decline (%)	P vs. TC	−6.0 [−7.9, −4.1]	−1.70 (v.large)	<0.001 *	
	P vs. TCP	−9.0 [−10.9, −7.1]	−2.55 (huge)	<0.001 *	
	TC vs. TCP	−3.0 [−4.6, −1.4]	−1.00 (large)	<0.001 *	

**Table 4:** (**a**) Technical and tactical performance (mean ± SD; 95% CI; ANOVA *p*-value; partial η^2^). (**b**) Pairwise between-group comparisons (Holm–Bonferroni-adjusted) for technical and tactical performance variables. The Abbreviations, Magnitude labels and Asterisk (*) explanations prefer to Table 1 footer.

(**a**)					
**Parameter**	**P (*n* = 27) M ± SD [95% CI]**	**TC (*n* = 27) M ± SD [95% CI]**	**TCP (*n* = 27) M ± SD [95% CI]**	***p* (ANOVA)**	**η^2^**
Passing accuracy (%)	75.0 ± 6.0 * [72.6, 77.4]*	82.0 ± 5.0 * [80.0, 84.0]*	84.0 ± 5.0 * [82.0, 86.0]*	<0.001	0.24
Accurate passes (*n*)	32.0 ± 6.0 * [29.6, 34.4]*	39.0 ± 5.0 * [37.0, 41.0]*	42.0 ± 5.0 * [40.0, 44.0]*	<0.001	0.21
Ball recoveries (*n*)	7.20 ± 2.00 * [6.41, 7.99]*	9.10 ± 2.10 * [8.27, 9.93]*	10.30 ± 2.20 * [9.43, 11.17]*	<0.01	0.17
Decision-making (1–10)	6.20 ± 1.00 * [5.80, 6.60]*	7.50 ± 0.90 * [7.14, 7.86]*	8.00 ± 0.80 * [7.68, 8.32]*	<0.001	0.26
Dribbling success (%)	62.0 ± 8.0 * [58.8, 65.2]*	70.0 ± 7.0 * [67.2, 72.8]*	73.0 ± 7.0 * [70.2, 75.8]*	<0.001	0.22
(**b**)					
**Parameter**	**Comparison**	**Mean Diff. [95% CI]**	**Cohen’s *d* (Mag.)**	***p* (Holm–Bonferroni)**	
Passing accuracy (%)	P vs. TC	−7.0 [−10.0, −4.0]	−1.27 (v.large)	<0.001 *	
	P vs. TCP	−9.0 [−12.0, −6.0]	−1.63 (v.large)	<0.001 *	
	TC vs. TCP	−2.0 [−4.7, 0.7]	−0.40 (small)	0.148	
Accurate passes (*n*)	P vs. TC	−7.0 [−10.0, −4.0]	−1.27 (v.large)	<0.001 *	
	P vs. TCP	−10.0 [−13.0, −7.0]	−1.81 (v.large)	<0.001 *	
	TC vs. TCP	−3.0 [−5.7, −0.3]	−0.60 (med.)	0.032 *	
Ball recoveries (*n*)	P vs. TC	−1.90 [−3.02, −0.78]	−0.93 (large)	0.003 *	
	P vs. TCP	−3.10 [−4.25, −1.95]	−1.47 (v.large)	<0.001 *	
	TC vs. TCP	−1.20 [−2.37, −0.03]	−0.56 (med.)	0.045 *	
Decision-making (1–10)	P vs. TC	−1.30 [−1.82, −0.78]	−1.37 (v.large)	<0.001 *	
	P vs. TCP	−1.80 [−2.29, −1.31]	−1.99 (v.large)	<0.001 *	
	TC vs. TCP	−0.50 [−0.97, −0.03]	−0.59 (med.)	0.036 *	
Dribbling success (%)	P vs. TC	−8.0 [−12.1, −3.9]	−1.06 (large)	<0.001 *	
	P vs. TCP	−11.0 [−15.1, −6.9]	−1.46 (v.large)	<0.001 *	
	TC vs. TCP	−3.0 [−6.8, 0.8]	−0.43 (small)	0.121	

**Table 5:** (**a**) Coaches’ evaluation of technical and tactical performance (mean ± SD; 95% CI; ANOVA *p*-value; partial η^2^). (**b**) Pairwise between-group comparisons (Holm–Bonferroni-adjusted) for coaches’ evaluation of technical and tactical performance. The Abbreviations, Magnitude labels and Asterisk (*) explanations prefer to Table 1 footer.

(**a**)					
**Parameter**	**P (*n* = 27) M ± SD [95% CI]**	**TC (*n* = 27) M ± SD [95% CI]**	**TCP (*n* = 27) M ± SD [95% CI]**	***p* (ANOVA)**	**η^2^**
Positioning (1–10)	6.50 ± 1.10 * [6.06, 6.94]*	7.60 ± 1.00 * [7.20, 8.00]*	8.20 ± 0.90 * [7.84, 8.56]*	<0.001	0.25
Off-ball movement (1–10)	6.30 ± 1.20 * [5.83, 6.77]*	7.40 ± 1.10 * [6.96, 7.84]*	8.00 ± 1.00 * [7.60, 8.40]*	<0.001	0.23
Support play (1–10)	6.70 ± 1.00 * [6.30, 7.10]*	7.80 ± 0.90 * [7.44, 8.16]*	8.30 ± 0.80 * [7.98, 8.62]*	<0.001	0.24
Pressing effectiveness (1–10)	6.40 ± 1.10 * [5.96, 6.84]*	7.30 ± 1.00 * [6.90, 7.70]*	7.90 ± 0.90 * [7.54, 8.26]*	<0.01	0.19
Tactical discipline (1–10)	6.80 ± 1.00 * [6.40, 7.20]*	7.70 ± 0.90 * [7.34, 8.06]*	8.10 ± 0.80 * [7.78, 8.42]*	<0.001	0.22
(**b**)					
**Parameter**	**Comparison**	**Mean Diff. [95% CI]**	**Cohen’s *d* (Mag.)**	***p* (Holm–Bonferroni)**	
Positioning (1–10)	P vs. TC	−1.10 [−1.67, −0.53]	−1.05 (large)	<0.001 *	
	P vs. TCP	−1.70 [−2.25, −1.15]	−1.69 (v.large)	<0.001 *	
	TC vs. TCP	−0.60 [−1.12, −0.08]	−0.63 (med.)	0.024 *	
Off-ball movement (1–10)	P vs. TC	−1.10 [−1.73, −0.47]	−0.96 (large)	0.002 *	
	P vs. TCP	−1.70 [−2.30, −1.10]	−1.54 (v.large)	<0.001 *	
	TC vs. TCP	−0.60 [−1.17, −0.03]	−0.57 (med.)	0.041 *	
Support play (1–10)	P vs. TC	−1.10 [−1.62, −0.58]	−1.16 (large)	<0.001 *	
	P vs. TCP	−1.60 [−2.09, −1.11]	−1.77 (v.large)	<0.001 *	
	TC vs. TCP	−0.50 [−0.97, −0.03]	−0.59 (med.)	0.036 *	
Pressing effectiveness (1–10)	P vs. TC	−0.90 [−1.47, −0.33]	−0.86 (large)	0.005 *	
	P vs. TCP	−1.50 [−2.05, −0.95]	−1.49 (v.large)	<0.001 *	
	TC vs. TCP	−0.60 [−1.12, −0.08]	−0.63 (med.)	0.024 *	
Tactical discipline (1–10)	P vs. TC	−0.90 [−1.42, −0.38]	−0.95 (large)	0.002 *	
	P vs. TCP	−1.30 [−1.79, −0.81]	−1.44 (v.large)	<0.001 *	
	TC vs. TCP	−0.40 [−0.87, 0.07]	−0.47 (small)	0.090	

## Data Availability

The data presented in this study are available on reasonable request from the corresponding author. The data are not publicly available due to confidentiality restrictions related to the participation of professional athletes.
